# Early Epidemic Dynamics of the West African 2014 Ebola Outbreak: Estimates Derived with a Simple Two-Parameter Model

**DOI:** 10.1371/currents.outbreaks.89c0d3783f36958d96ebbae97348d571

**Published:** 2014-09-08

**Authors:** David Fisman, Edwin Khoo, Ashleigh Tuite

**Affiliations:** Dalla Lana School of Public Health, University of Toronto, Toronto, Ontario, Canada; Dalla Lana School of Public Health, University of Toronto, Toronto, Ontario, Canada; Dalla Lana School of Public Health, University of Toronto, Toronto, Ontario, Canada

## Abstract

The 2014 West African Ebola virus outbreak, now more correctly referred to as an epidemic, is the largest ever to occur. As of August 28, 2014, concerns have been raised that control efforts, particularly in Liberia, have been ineffective, as reported case counts continue to increase. Limited data are available on the epidemiology of the outbreak. However, reported cumulative incidence data as well as death counts are available for Guinea, Sierra Leone, Liberia and Nigeria. We utilized a simple, two parameter mathematical model of epidemic growth and control, to characterize epidemic growth patterns in West Africa, to evaluate the degree to which the epidemic is being controlled, and to assess the potential implications of growth patterns for epidemic size. Models demonstrated good fits to data. Overall basic reproductive number (R0) for the epidemic was estimated to be between 1.6 and 2.0, consistent with prior outbreaks. However, we identified only weak evidence for the occurrence of epidemic control in West Africa as a whole, and essentially no evidence for control in Liberia (though slowing of growth was seen in Guinea and Sierra Leone). It is projected that small reductions in transmission would prevent tens of thousands of future infections. These findings suggest that there is an extraordinary need for improved control measures for the 2014 Ebola epidemic, especially in Liberia, if catastrophe is to be averted.

## Introduction

Ebola virus is a zoonotic filovirus that causes a hemorrhagic fever syndrome in humans, with a high case-fatality rate 5. An epidemic that started in December 2013 or early in 2014 6 , and which has spread to four West African countries (Guinea,Liberia, Sierra Leone, and Nigeria), has infected over 2000 individuals as of August 28 2014 8 . While Ebola virus has caused several prior outbreaks in Africa, this is the largest ever to occur 7 . Over half of all Ebola infections that have *ever* been documented have occurred in the context of the current outbreak, which is ongoing.

In addition to a case-fatality rate greater than 50%, the current West African outbreak has proven difficult to control, has resulted in international travel advisories, flight cancellations and border closures 12 , and has led to episodes of civil unrest, particularly in Liberia 11 ^,^ 10 . The occurrence of cases in very large, well-connected urban centers (including capitol cities of all affected countries) has fueled concerns about the international spread of disease via air travel, and on August 8, the World Health Organization declared the outbreak to be a Public Health Emergency of International Concern 9. This, again, contrasts with earlier African Ebola outbreaks which have mostly occurred in smaller and less-connected cities, towns and rural areas 7 .

Mathematical models of infectious disease outbreaks and epidemics can be useful tools for synthesizing available information on an infectious disease process, transforming data into useable knowledge, and defining and quantifying uncertainty about infectious diseases^ 13^^,^ 14. Many models are explicitly "mechanistic" and represent epidemics as processes that result in transition of individuals in the population between health states (e.g., susceptible, infectious, and immune) 13 ^,^ 14 . However, construction of such models requires fairly detailed information on incidence, immune status in the population, and contact patterns for correct parameterization. We have recently described a very simple mathematical model (the Incidence Decay with Exponential Adjustment, or "IDEA", model) that can be used as a descriptive and prognostic tool for epidemic processes when only limited data (e.g., cumulative incidence curves) are available 1 . Here we apply this model to this epidemic; our objectives are to reproduce, mathematically, observed patterns of epidemic growth, and to gain insights into the degree to which current control efforts are likely to impact epidemic size and duration.

## Methods

Data Sources

Case data, including cumulative incidence, and cumulative deaths, by date of report, for Liberia, Sierra Leone, Guinea, and Nigeria were obtained from a public data repository maintained by Caitlin Rivers of Virginia Polytechnic Institute (https://github.com/cmrivers/ebola). These data are derived from official case counts from the World Health Organization, but have been aggregated and organized, making them an efficient resource for model fitting. Total case counts from this source do not distinguish between suspect, probable and confirmed case counts, and consequently cumulative incidence may decrease between measurements reflecting suspect cases who have been excluded through testing or other means. As only a fraction of cases are subject to virological confirmation, we also obtained virologically-confirmed case counts, by date, from the Virology Down Under blog, maintained by Dr. Ian Mackay (http://virologydownunder.blogspot.com.au/). These estimates are, again, derived from World Health Organization reports. Dr. Mackay's graphs are created in the Tableau application, and numerical data can be obtained using Tableau (http://www.tableausoftware.com).

Model

We utilized the previously described "incidence decay with exponential adjustment" (IDEA) model to evaluate epidemic dynamics 1 . This model describes epidemic processes both in terms of exponential growth (a function of the basic reproductive number, R_0_) and in terms of simultaneous decay, brought about by behavioral change, public health interventions, increased immunity in the population, or any other dynamic change that slows disease transmission; the model is descriptive and cannot distinguish between putative controlling mechanisms, but has the advantage of allowing epidemic growth to slow even before the critical fraction of susceptibles in the population is exhausted 1 ^,^ 13 . We have previously shown excellent agreement between the IDEA model and a discrete-time susceptible-infectious-removed (SIR) compartmental model, when R_0_ is low or moderate 1 . The IDEA model has several attractive properties: it can be readily parameterized by fitting to either incidence or cumulative incidence data, requires no assumptions regarding immune status in the population, and appears to provide reasonably accurate projections about epidemic size and duration (in the absence of change in control efforts) based on pre-peak epidemic data when R_0_ is low or moderate. Furthermore, comparison with simulations suggests that the model can identify multi-wave epidemics or abrupt changes in control based on sudden changes in the value of the control parameter *d *(as described below) from generation to generation^1^.

The model utilizes the following functional form: \begin{equation*}I_{t} =((R_0/(1+d)^t)^t\end{equation*}, where *t *is scaled in generation time, with R_0 _the basic reproductive number, and *d* a "control parameter" that causes incidence to decay. I_t _represents incident cases in a given generation. In the absence of control, incident case counts grow to the power of *t*. However, when control is present, the effective reproduction number is reduced by a power of *t^2^*, causing transmission to slow and stop even when the absolute value of *d *is small. Best fit parameter values are estimated by fitting; we have generally identified preferred parameter values as those that minimize the root-mean-squared distance between model estimates and empirical data, but other approaches (e.g., Bayesian maximum likelihood approaches) are also possible.

Analyses

We utilized prior estimates of incubation period for Ebola virus infection (mean approximately 13 days) 3 and duration of infectivity (estimated at 3-5 days) 2 to derive a generation time of 15 days for the outbreak, based on the heuristic *t* = incubation + 1/2 infective period, though this was varied (from 12 days to 18 days) in sensitivity analyses. We assumed incubation to be equivalent to latency for this virus. The initial reported case count was 42 cases on March 22, 2014. Prior reports of R_0_ for Ebola virus have ranged from 1.5 to 2.7 2 ^,^ 4 , and this cumulative case count would be consistent with that seen in approximately the fifth generation of an epidemic process with R0 ~ 2; this was, again, varied from 3 generations to 7 generations in sensitivity analyses.

In our base case analyses, we fit our model to time series data iteratively, using a progressively increasing number of outbreak generations. Fits were performed using the Berkeley Madonna software package (Berkeley, California, http://www.berkeleymadonna.com/), both using the built-in "curve fit" function, and also by evaluating root-mean-squared distances between model estimates and observations for varying combinations of R_0_ and *d*.

Model fits utilized epidemic time series available as of August 22, 2014. In addition to fitting models to overall epidemic cumulative incidence curves, we fit models to country-specific data from Guinea, Liberia, and Sierra Leone. A separate model was not fitted to data from Nigeria due to the low case count (N = 15) at the time of writing. For the purposes of fitting country-level models, we used the same estimated start date for the Guinea epidemic as was used for the outbreak overall, while the first generation of Liberia's outbreak was dated to March 27, 2014, and Sierra Leone's to May 27, 2014.

## Results

Base Case Analysis

As increasing numbers of outbreak generations were used, best fit R_0_ estimates and estimates of *d *declined (Figure 1). We identified no abrupt surges in *d *that simulations suggest are indicative of multi-wave outbreaks 1 . There was a range of combinations of R_0 _and d that provided approximate fits to observed case counts, but RMSD was lowest, by an order of magnitude, for R_0 _values close to 1.8, and d values close to 0.01 (Figure 2). Our best fit model identified Ro as 1.78, and d as 0.009. Cumulative model case counts were projected to be 2435 as compared to 2473 observed cases (Figure 3).

Based on these parameter values, and in the absence of increase in *d* as a result of intervention, the outbreak would be projected to have caused over 25,000 infections by the end of 2014. A peak in the epidemic would not occur until April 2015, and continue until mid-2016, with a final size greater than 140,000 cases. However, epidemic size and duration are projected to be extremely sensitive to incremental increases in the size of d. For example, a September 2014 increase in d by 0.005 (to 0.014 rather than 0.009) would diminish the projected epidemic size to < 10,000 cases, with incidence steadily diminishing rather than increasing in the coming months.

Sensitivity Analyses and Alternate Approaches

We also fit separate models to epidemic curves derived from reported deaths, rather than cases, curves based only on virologically confirmed cases, as well as curves based on varying assumptions about case-under-reporting, epidemic duration prior to first reporting in March 2014, and generation times. None of these analyses provided estimates of R_0_ and *d* that differed markedly from those derived in the base case (Table 1), though it should be noted that the exponential nature of epidemic growth means that even small changes in model parameters would result in large differences in final epidemic sizes.

In our base case we fitted IDEA models to overall cumulative epidemic curves, but fitting curves to individual country-level epidemics (Figure 5), and summing these curves, also reproduced the overall epidemic curve well (Figure 6). However, the epidemic dynamics of individual countries were quite distinct from one another. In Liberia, a low R_0 _with no control (*d* ~ 0) contrasted with a high R_0_ and high control (*d* = 0.22) in Sierra Leone. Model projections suggested that as of August 2014 Sierra Leone and Guinea's outbreaks are slowing, whereas the Liberian outbreak continues to grow in an exponential manner.

**Parameter Estimates by Generation of Epidemic:**
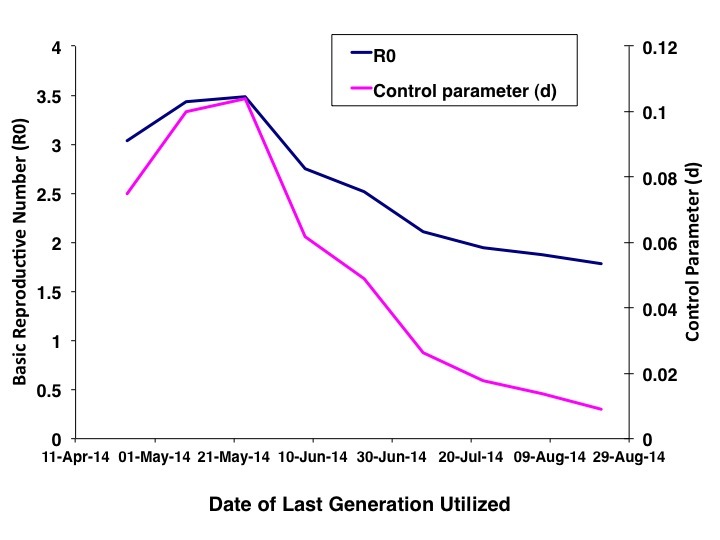
The graph plots best fit values of R_0_ (blue curve) and *d* (pink curve) for the IDEA model obtained by utilizing progressively increasing numbers of epidemic generations (figure shows fits for generation 7 through 15).

**Contour Plot of Model Fit as a Function of R0 and d.**
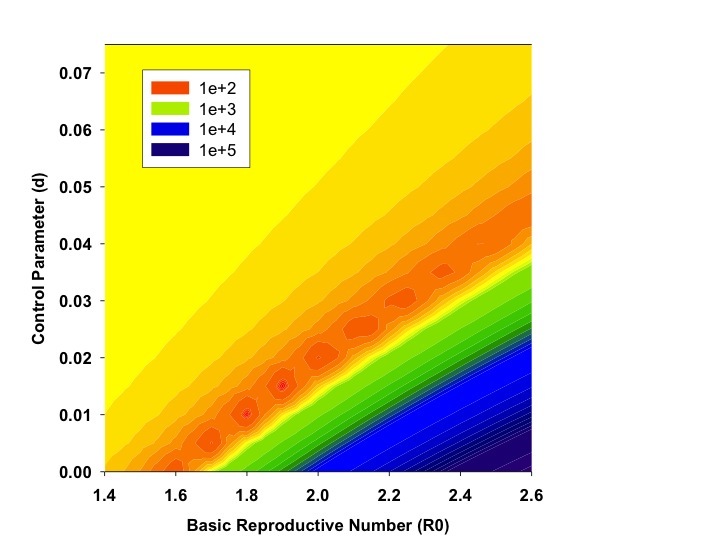
Figure plots root mean squared distance (RMSD) of model projected case counts from observed case counts in models utilizing varying combinations of R0 (X-axis) and *d* (Y-axis). Dark orange areas signifying lowest RMSD are seen with R0 in the 1.75-1.90 range, and with *d* from 0.01 to 0.015.

**Overall Observed vs. Expected Cumulative Incidence:**
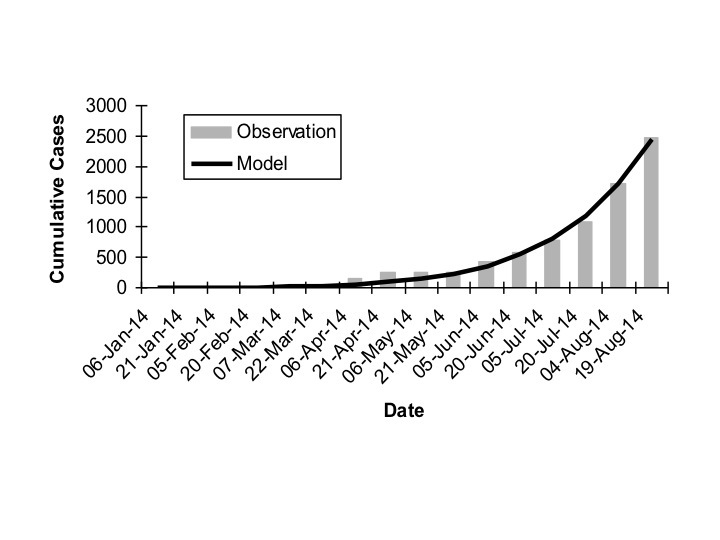
Best fit model (dark curve) (R0 = 1.78, d = 0.009) to observed cumulative incidence for West Africa by generation (gray bars). A 15 day serial interval is assumed, and first reported cases are assumed to have been reported in generation 5.

**Projections of Incidence and Cumulative Incidence to January 1, 2015:**
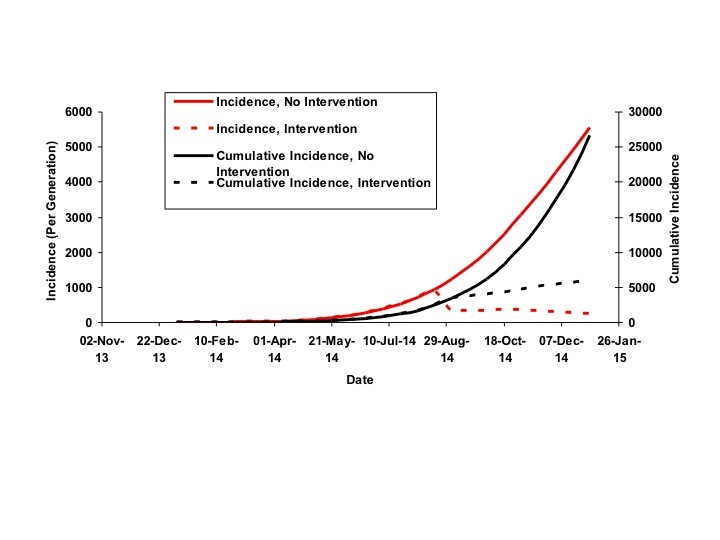
The figure plots model-projected incidence (per 15-day generation) (solid red curve, scale on left Y-axis) and cumulative incidence (solid black curve, scale on right Y-axis) against time (X-axis). Dashed curves show the potential impact of intervention in September 2014 on incidence (dashed red curve) and cumulative incidence (dashed black curve), if intervention resulted in an increase of d by 0.005.

**Country Specific Model Fits, Observed vs. Expected Cumulative Incidence:**
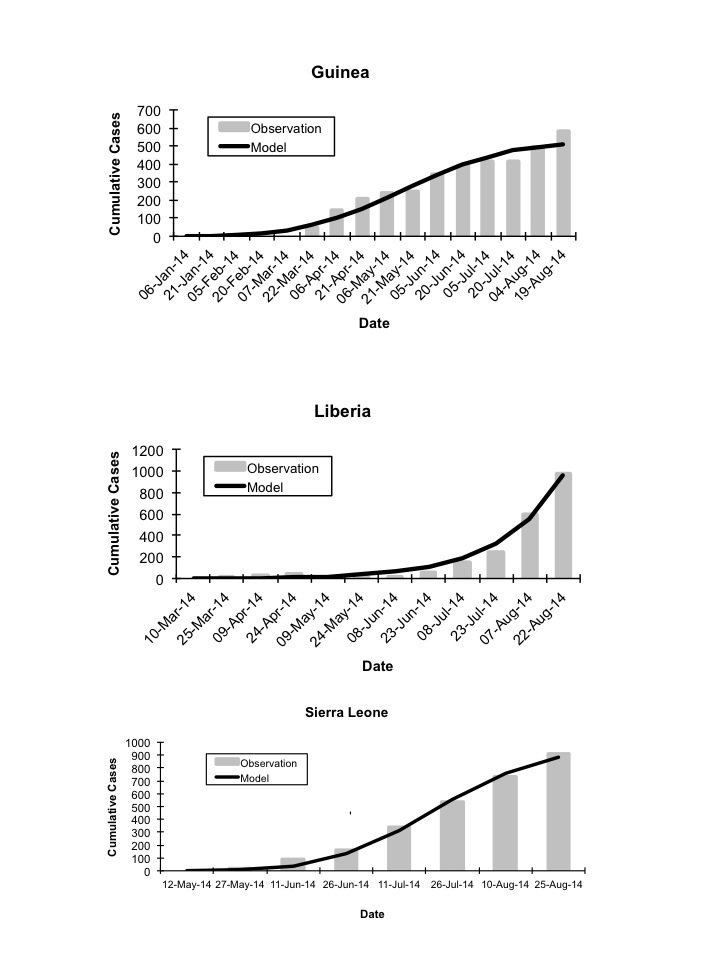
Graphs demonstrate good model fits (dark curves) to observed generation by generation cumulative incidence of infection in Guinea (top panel), Liberia (middle panel), and Sierra Leone (bottom panel).

**Overall vs. Country-Level Model Fits:**
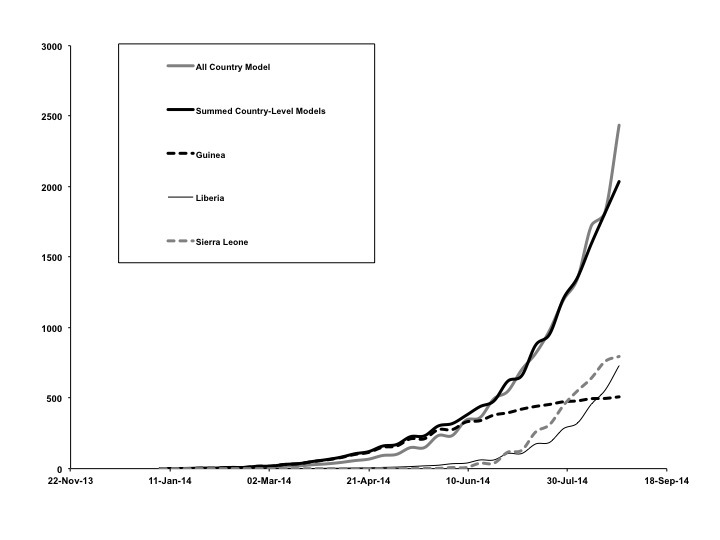
Graphs show good agreement between the base-case model, fit to overall cumulative incidence data (all countries combined, solid gray curve) vs. summed outputs (solid black curve) from models fit to country-level data from Guinea (dashed black curve), Liberia (thin black curve), and Sierra Leone (dashed gray curve).

**Table 1. Sensitivity of Model Estimates to Alternate Approaches and Assumption:** 

Alternate Assumption	*R_0_*	*d*
Base case	1.78	0.009
12 day generation time	1.68	0.009
18 day generation time	1.94	0.013
Outbreak recognized generation 3	2.19	0.022
Outbreak recognized generation 7	1.70	0.011
Outbreak 50% under-reported	1.92	0.013
Outbreak 100% under-reported	2.02	0.015
Virologically confirmed cases only	1.74	0.011
Deaths only	1.66	0.008
Guinea cases only	2.46	0.050
Liberia cases only	1.72	0
Sierra Leone cases only	8.33	0.22

## Discussion

The 2014 Ebola epidemic now stands as the largest ever recorded, and threatens not only health and healthcare institutions, but civil institutions, in affected countries. Based on models fit to available cumulative incidence data from August 2014, we project that in the absence of more effective control interventions, this epidemic will increase to affect tens, and possibly hundreds, of thousands of individuals. Given the high case fatality ratio associated with Ebola virus infection, such an occurrence would be nothing short of catastrophic. Based on data currently available to us, it appears that this threat is currently centered on the Liberian component of the epidemic, which can be characterized as a simple exponential growth process, with little evidence for slowing of transmission. This contrasts with outbreaks in Guinea and Sierra Leone.

The IDEA model is descriptive, and consequently it is not possible to attribute mechanisms to the "decay" parameter (d) which defines slowing of growth. In more complex and explicit models, the effects that occur via *d* could occur with decreased rates of contact with infectious individuals, greater availability of personal protective items, behavioral change, depletion of susceptibles, or any other factor that impacts the effective reproductive number or force of infection of an epidemic process 13 ^,^ 14. Nonetheless, this parameter describes the observed tendency of epidemics and outbreaks to end before the critical density of susceptibles is exhausted 1. In our published 1 , and as yet unpublished applications of this modeling approach to influenza and MERS coronavirus epidemics and outbreaks, negligible values for *d*, as observed in Liberia, have been distinctly unusual. However, uninterrupted exponential growth of an epidemic, as we observe in Liberia, is consistent with media reports and communication from colleagues in the field. We project that even small, incremental near-term increases in control (as defined by *d*) would result in thousands or tens of thousands of infections prevented. The value of 0.005 used in our analysis is arbitrary; unfortunately, the novelty of the IDEA approach means that we have very limited understanding of the empiric size of real-world control factors that would translate to a given change in *d.* However, our experiment confers a control factor on Liberia less than that that appears to be operative in Guinea, so we believe this is likely to represent an attainable increase in control.

As with any mathematical model, ours is limited by the quality of data used for model calibration. Numerous factors, including limited resources, understandable concerns for personal safety among healthcare and public health personnel, civil unrest, and limited virological resources, are likely to combine to make accurate enumeration of cases difficult. We performed numerous sensitivity analyses, and found that use of deaths or virologically confirmed case numbers, variation in plausible starting date or generation time, and varying assumptions about (constant) under-reporting resulted in very little change in best-fit model parameters. That said, factors such as abrupt decreases or surges in case reporting (as opposed to occurrence) would be likely to result in distortion of model-based estimates.

For almost all scenarios evaluated, and for all countries except for Sierra Leone evaluated by single-country models, we found estimates of R_0 _similar to those that have been reported previously for Ebola outbreaks 2 ^,^ 4 . For Sierra Leone, we found an outbreak that appeared to explode extremely rapidly, and then be controlled with similar rapidity. Whether this reflects actual disease dynamics or artifacts of disease surveillance and reporting remains to be seen.

## Conclusion

Using a simple, two-parameter mathematical model, we find that the initial growth characteristics of the 2014 West African Ebola epidemic to be similar to those associated with prior Ebola outbreaks. Concerning is the lack of control evident, with epidemic processes growing in an essentially uncontrolled exponential manner, particularly in Liberia. While further data will permit model validation or re-calibration in the coming months, our findings indicate that this epidemic represents a public health emergency which has the potential to grow to extraordinarily destructive dimensions. We hope our model will add support to those voices already calling for an extraordinary international cooperative effort to control this epidemic.

## Competing Interests

The authors have declared that no competing interests exist.
