# Supplementary material for: Early Epidemic Dynamics of the West African 2014 Ebola Outbreak: Estimates Derived with a Simple Two-Parameter Model
Source: PLoS Curr. 2014 Sep 8;6:ecurrents.outbreaks.89c0d3783f36958d96ebbae97348d571. [Version 1] doi: 10.1371/currents.outbreaks.89c0d3783f36958d96ebbae97348d571 (PMC4169344; doi:10.1371/currents.outbreaks.89c0d3783f36958d96ebbae97348d571)
Supplement: Related texts

# Reply

**Published:** 2014-09-15

**Authors:** David Fisman

**Affiliations:** University of Toronto

Were you looking for this paper? http://currents.plos.org/outbreaks/article/assessing-the-international-spreading-risk-associated-with-the-2014-west-african-ebola-outbreak/ 

# Reply

**Published:** 2014-09-15

**Authors:** eryan

Where is the part that indicates the top 16 countries Ebola may spread to by percentage? 

# Reply

**Published:** 2014-09-09

**Authors:** Dominic Della Sera

Good stuff, this is what CNN needs to show.
